# Mechanism and effects of STING–IFN-I pathway on nociception: A narrative review

**DOI:** 10.3389/fnmol.2022.1081288

**Published:** 2023-01-04

**Authors:** Jinghan Yang, Hui Ding, Bo Shuai, Yan Zhang, Yan Zhang

**Affiliations:** ^1^Department of Anesthesiology, Union Hospital, Tongji Medical College, Huazhong University of Science and Technology, Wuhan, China; ^2^Department of Obstetrics and Gynecology, Union Hospital, Tongji Medical College, Huazhong University of Science and Technology, Wuhan, China; ^3^Department of Integrated Traditional Chinese and Western Medicine, Union Hospital, Tongji Medical College, Huazhong University of Science and Technology, Wuhan, China; ^4^Department of Pain, Union Hospital, Tongji Medical College, Huazhong University of Science and Technology, Wuhan, China; ^5^Institute of Anesthesia and Critical Care Medicine, Union Hospital, Tongji Medical College, Huazhong University of Science and Technology, Wuhan, China

**Keywords:** nociception, stimulator of interferon genes, interferon-I, peripheral nerve system (PNS), central nerve system (CNS)

## Abstract

Since the discovery of STING in 2008, numerous studies have investigated its functions in immunity, inflammation, and cancer. STING activates downstream molecules including IFN-I, NLRP3, and NF-κB. The STING–IFN-I pathway plays a vital role in nociception. After receiving the upstream signal, STING is activated and induces the expression of IFN-I, and after paracrine and autocrine signaling, IFN-I binds to IFN receptors. Subsequently, the activity of ion channels is inhibited by TYK2, which induces an acute antinociceptive effect. JAK activates PIK3 and MAPK–MNK–eIF4E pathways, which sensitize nociceptors in the peripheral nervous system. In the mid-late stage, the STING–IFN-I pathway activates STAT, increases pro-inflammatory and anti-inflammatory cytokines, inhibits ER-phagy, and promotes microglial M1-polarization in the central nervous system, leading to central sensitization. Thus, the STING–IFN-I pathway may exert complex effects on nociception at various stages, and these effects require further comprehensive elucidation. Therefore, in this review, we systematically summarized the mechanisms of the STING–IFN-I pathway and discussed its function in nociception.

## Introduction

Pain is defined by the International Association for the Study of Pain as an unpleasant sensory and emotional experience associated with actual or potential tissue or other damage (Merskey and Spear, 1967). After sensing physical and chemical stimuli, nociceptors produce and transmit information to the central nervous system(CNS). Notably, the brain can generate pain without a message from nociceptors or the spinal cord, such as in phantom limb pain (Loeser and Melzack, 1999; Julius and Basbaum, 2001). Multiple molecules are involved in the production of pain, such as G protein-coupled receptors, cyclic nucleotides, capsaicin, and acid (Julius and Basbaum, 2001).

Stimulator of interferon genes (also called *STING*, *MITA*, *MPYS*, *ERIS*, and *TMEM173*) was first discovered in 2008 (Ishikawa and Barber, 2008; Zhong et al., 2008). STING could regulate antimicrobial response, autoimmune disease, and cancer progression (Ahn et al., 2012; Li et al., 2013; Zheng et al., 2020). The stimulator of interferon genes (STING)–interferon-I (IFN-I) pathway can control nociception (Donnelly et al., 2021; Wang et al., 2021). In neuropathic pain models including bone cancer pain, chemotherapy-induced peripheral neuropathy, and nerve injury, administration of STING agonists activates STING, increases the expression of IFN-I, and inhibits the excitability of nociceptors in the peripheral nervous system (PNS) (Wang et al., 2021). These effects induce transient, short-term, and dose-dependent antinociception at an early stage (Donnelly et al., 2021). However, the antinociceptive effect was not substantial 11 days after the injection (Wang et al., 2021). Similarly, activation of the STING–IFN-I pathway induces nociception or neuropathic pain at a late stage (Liu et al., 2022; Wu et al., 2022). The exact effects of STING–IFN-I remain controversial, and its differential role in different sexes, neuropathic pain models, cells, and stages requires further research. Previous studies hypothesized that this pathway might be a potential therapeutic target for pain management.

In this review, we systematically summarize the mechanisms of the STING–IFN-I pathway and discuss its function in nociception.

## Structure and properties of STING and IFN-I

STING is located in the endoplasmic reticulum (ER) (Ishikawa and Barber, 2008; Zhong et al., 2008). In human cells, STING comprises 379 amino acids and contains five putative transmembrane regions (Ishikawa and Barber, 2008). The N-terminal of STING, consisting of four transmembrane regions, is responsible for membrane anchoring. The C-terminal protrudes into the cytoplasm and contains a domain that binds with cyclic dinucleotides (CDNs) (Zhong et al., 2008; Sun et al., 2009; Landman et al., 2020). STING can directly detect bacterial CDNs and activate immune responses (Burdette et al., 2011; Ablasser et al., 2013). In addition, it can detect cytosolic double-stranded DNA (dsDNA) released by tumor and dead cells *via* cyclic guanosine monophosphate–adenosine monophosphate (cyclic GMP–AMP or cGAMP) synthase (cGAS) activity (Chen et al., 2016). Moreover, leakage of mitochondrial DNA can activate STING in adjacent phagocytic cells (West et al., 2015). After STING activation, the expression of IFN-I, NOD-like receptor protein 3 (NLRP3), and nuclear factor-κB (NF-κB) increases (Zhong et al., 2008; Abe and Barber, 2014; Liu et al., 2015).

IFN-I was first discovered in 1957 and is composed of IFN-α, IFN-β, IFN-δ, IFN-ε, IFN-κ, IFN-τ, and IFN-ω (Lindenmann et al., 1957; Tan et al., 2021). IFN-I participates in the antiviral response, cell proliferation, apoptosis, inflammation, and adaptive immunity (Lindenmann et al., 1957; Stark et al., 1998; Randall and Goodbourn, 2008).

## Research progress of STING–IFN-I pathway

### Antimicrobial response

Microbial DNA invasion triggers a series of immune responses. STING is essential for detecting exogenous microbial DNA (Li et al., 2013). Activation of STING consequently activates the transcription factors NF-κB and interferon regulatory factor 3 (IRF3) to induce cytokines and IFN-I expression (Ishikawa and Barber, 2011). STING is required by fibroblasts, macrophages, dendritic cells, and myeloid cells to induce IFN-I production against vaccinia virus (VACV), cytomegalovirus (HCMV), baculovirus, several strains of herpes simplex virus-1 (HSV1), and *Listeria monocytogenes* (Ishikawa and Barber, 2008; Ishikawa et al., 2009).

### Autoimmune disease

In addition to exogenous DNA, STING can detect self-DNA. Undigested DNA from apoptotic cells triggers DNA sensors, which increase the expression of cytokines and result in autoimmune diseases (Nagata, 2010; Ahn et al., 2012). The exonuclease, three prime repair exonuclease 1 (TREX1), degrades cytosolic DNA (Mazur and Perrino, 1999; Crow et al., 2006) and its deficiency leads to multiple inflammatory and autoimmune diseases such as systemic lupus erythematosus, Aicardi–Goutieres syndrome, and familial chilblain lupus (Rice et al., 2015). In a TREX1-deficient rat model, cGAS activated STING through cGAMP production and mediated inflammatory disease and death in mice (Gao et al., 2015). Similarly, STING triggered by apoptotic or necrotic DNA promoted the expression of cytokines, whereas its deficiency abrogated the production of cytokines activated by self-DNA in a DNase II-deficient model (Ahn et al., 2012).

### Cancer progression

Nuclear and mitochondrial DNA are easily damaged in tumor cells, inducing IFN-I through the cGAS–STING–IRF3-dependent pathway (Woo et al., 2014; Chen Y. A. et al., 2017; Mackenzie et al., 2017). IFN-I is a mediator of STING and exerts adaptive antitumor effects (Zheng et al., 2020). It can promote cross-presentation by stimulating the maturation of DCs, slowing down the endosome–lysosome acidification process to prevent phagocytic tumor antigen clearance, and increasing the expression of cell surface MHC I molecules, which accelerates DC migration to lymph nodes to cross-trigger tumor-specific CD8^+^ T cells (Reboulet et al., 2010; Diamond et al., 2011; Lorenzi et al., 2011; Zheng et al., 2020). In addition, IFN-I can induce the expression of multiple chemokines (Padovan et al., 2002; Takashima et al., 2016). For instance, CXCL9 and CXCL10 are involved in cytotoxic T lymphocyte transfer and infiltration, whereas CCL5 and CXCL10 promote the recruitment and activation of NK cells and T cells in tumors (Padovan et al., 2002; Takashima et al., 2016). By contrast, the cGAS–STING pathway can induce the senescence-associated secretory phenotype (SASP) (Loo et al., 2020). The SASP factor induces immune surveillance and acts as a tumor suppressor. However, continuous exposure to SASP may cause tissue damage and chronic inflammation associated with tumor growth (Loo et al., 2020). Nevertheless, long-term activation of STING may promote tumor growth and metastasis, and this effect is associated with tumor stage, CIN status, and the degree of STING activation (Zheng et al., 2020). STING agonists including cyclic dinucleotides and their derivatives, DMXAA and its analogs, and small-molecule agonists are widely studied as cancer treatment agents (Corrales and Gajewski, 2015; Ablasser and Chen, 2019; Zheng et al., 2020).

## Mechanism of STING–IFN-I pathway with respect to pain

### Peripheral nociceptors and pain

Cell bodies of nociceptors are distributed in the dorsal root ganglia (DRG) and trigeminal ganglion (Basbaum et al., 2009). Most nociceptors contain unmyelinated C fibers (Woolf and Ma, 2007). However, initial and acute pain is mediated by nociceptors with A fibers (Djouhri and Lawson, 2004). After sensing physical and chemical stimuli, peripheral nociceptors are activated to produce pain through different signal transduction pathways (Julius and Basbaum, 2001; Donnelly et al., 2020). Particularly, TRP channels recognize noxious heat, and the ENaC/DEG channel family senses mechanical stimuli (Lingueglia et al., 1997; Tominaga et al., 1998). Nociceptors can convert receptor potentials into action potentials through voltage-gated channels (including sodium, calcium, and potassium channels) (Dubin and Patapoutian, 2010). Primary nociceptors transmit noxious stimuli to projection neurons located in the cornu dorsalis medullae spinalis (Basbaum et al., 2009). Harmful information is transmitted to the somatosensory cortex through the thalamus, indicating the location and intensity of the pain (Basbaum et al., 2009). Other projection neurons contact the cingulate gyrus and insular cortex through the brain stem and amygdala, forming emotional elements of pain experiences (Basbaum et al., 2009).

### Pattern recognition receptors in nociception

**FIGURE 1:**
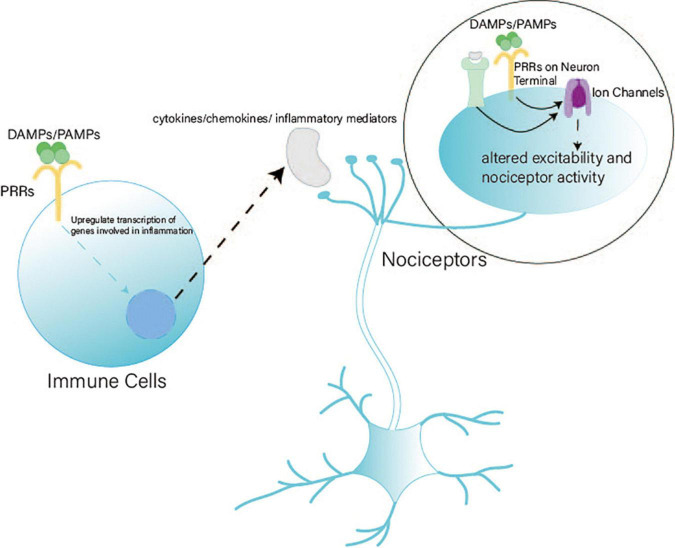
Pattern recognition receptors (PRRs) in nociception. PRRs play a vital role in nociception through indirect and direct pathways. (1) Indirect pathway: After PRRs on immune cells detect DAMPs/PAMPs, immune cells release cytokines, chemokines, and inflammatory mediators to react with the terminals of nociceptors. (2) Direct pathway: Terminals of nociceptors express PRRs to detect DAMPs/PAMPs directly. Indirect and direct pathways can alter the excitability and activity of ion channels to regulate nociceptors.

Pattern recognition receptors (PRRs) recognize pathogen-associated molecular patterns (PAMPs) and damage-associated molecular patterns (DAMPs) to induce the transcription of genes involved in inflammatory responses (Takeuchi and Akira, 2010). PRRs include toll-like, RIG-I-like, NOD-like, and DNA receptors (cytosolic sensors for DNA) (Takeuchi and Akira, 2010; Kumar et al., 2011). Both immune cells and peripheral nociceptors express PRRs (Usoskin et al., 2015; Zeisel et al., 2018; Zheng et al., 2019). Cytosolic DNA sensors such as the cGAS–STING pathway are highly expressed in nociceptive neurons (Usoskin et al., 2015; Zeisel et al., 2018; Donnelly et al., 2020). PRRs on immune cells recognize DAMPs/PAMPs and release cytokines/chemokines and inflammatory mediators to react with nociceptor terminals (Donnelly et al., 2020). Meanwhile, the terminals of nociceptors can directly detect PAMPs/DAMPs and danger signals (Donnelly et al., 2020). The indirect and direct pathways can regulate the function of sodium (e.g., Na_*v*_1.7, Na_*v*_1.8, and Na_*v*_1.9), calcium, and transient receptor potential channels. Thus, the excitability and activity of nociceptors are altered (Jin, 2006; Gold and Gebhart, 2010; Ji et al., 2014; Figure 1).

### Upstream signals of STING

**FIGURE 2:**
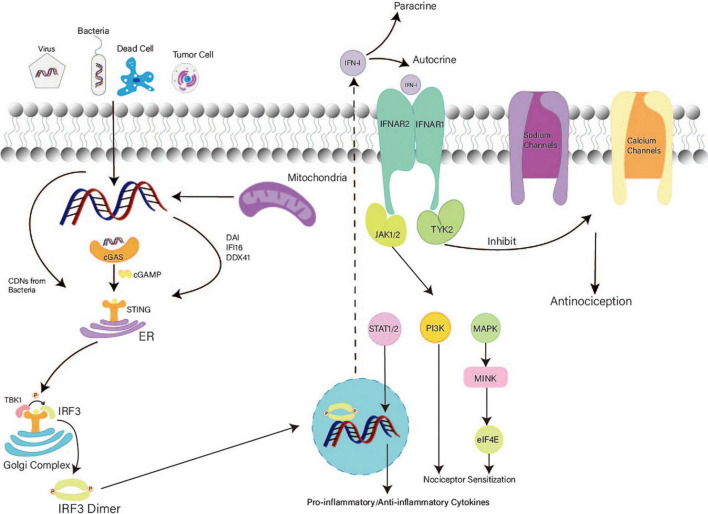
Mechanism of the STING–IFN-I pathway in nociception. After detecting both foreign and autologous cytosolic DNAs, STING exposes its C terminal and recruits TBK1. Activated STING transfers from the ER to the Golgi complex and recruits IRF3. IRF3 phosphorylated by TBK1 forms dimers and accesses the nucleus to activate the transcription of IFN. Secretion of IFN-I increases. Then, IFN-I binds with IFN receptors. TYK2 associated with IFNAR1 promotes the acute antinociceptive effect. JAK associated with IFNAR2 activates the MAPK–MNK–eIF4E pathway and PIK3 at a later stage, causing nociceptor sensitization in DRG. In the mid-late stage, IFN-I induces a delayed effect in the CNS.

cGAS, a cytosolic sensor for DNA, can activate STING through cGAMP production (Ablasser et al., 2013; Gao et al., 2013; Li et al., 2013; Sun et al., 2013). STING directly detects bacterial cytoplasmic CDNs including cyclic-di-GMP, cyclic-di-AMP, and 3′,3′-cGAMP (Burdette et al., 2011; Jin et al., 2011; Ablasser et al., 2013; Yi et al., 2013). Aside from cGAS, DNA-dependent activators of interferon regulatory factors, IFN-γ-inducible protein 16, and DEAD box polypeptide 41 can also recognize cytosolic DNA and activate STING (Tanaka and Chen, 2012; Wu and Chen, 2014; Cheng et al., 2020; Figure 2). Intracellular dsDNA and dsRNA can induce IFN-I-dependent antinociception; however, only the dsDNA-dependent pathway requires the cGAS–STING pathway (Donnelly et al., 2021).

### Downstream signals of STING

After binding with CDNs, STING is transferred from the ER to the Golgi complex *via* perinuclear vesicles (Ishikawa et al., 2009). STING forms oligomers in the ER–Golgi membrane and exposes its C terminal (Tanaka and Chen, 2012; Ergun et al., 2019). The C terminal of STING recruits TANK-binding kinase 1 (TBK1), and the STING dimer accesses the active site of TBK1 for its phosphorylation (Barber, 2011; Zhang et al., 2019). In addition, two TBK1 dimers can be mutually activated *via* transautophosphorylation (Zhang et al., 2019). The phosphorylated tail of STING recruits IRF3 and transports it to TBK1 for phosphorylation (Tanaka and Chen, 2012; Zhang et al., 2019). Notably, the interaction between STING and TBK1 enhances the binding of TBK1 and IRF3 (Zhong et al., 2008; Tanaka and Chen, 2012; Zhang et al., 2019). Phosphorylated IRF3 dimers access the nucleus and activate the transcription of IFN and inflammatory factor genes (Liu et al., 2015; Zhang H. et al., 2020). Thus, IFN-I synthesis increases notably. After paracrine and autocrine signaling, IFN-I binds with IFN receptors on sensory neurons to generate nociceptive effects (Donnelly et al., 2021; Tan et al., 2021). IFN receptors comprise IFNAR1 and IFNAR2 (Wang et al., 2021). IFNAR1 plays a vital role in acute nociceptive functions (Donnelly et al., 2021; Wang et al., 2021). Inhibition of tyrosine kinase (TYK2) eliminates the analgesic effect of IFN-β (Donnelly et al., 2021). Thus, TYK2 associated with IFNAR1 inhibits the activity of sodium (Na_*v*_1.7) and calcium channels (Donnelly et al., 2021; Tan et al., 2021). Furthermore, the low excitability of nociceptors is attributed to the loss of function of sodium (Na_*v*_1.7) and calcium channels (Binshtok et al., 2007; McDermott et al., 2019). Therefore, IFN-I can induce acute and short-term antinociception *via* TYK2.

Conversely, IFNAR2 is associated with Janus-activated kinases (JAKs) (Michalska et al., 2018; Tan et al., 2021). After activation of JAKs, the mitogen-activated protein kinase (MAPK)–interacting kinase (MNK)–eukaryotic translation initiation factor 4E (eIF4E) pathway and PIK3 are activated, causing nociceptor sensitization in the PNS at a later stage (Tan et al., 2021). In the mid-late stage, IFN-I activates STAT to induce the expression of pro-inflammatory and anti-inflammatory cytokines, inhibits ER-phagy, and promotes microglial M1-polarization, which generates delayed nociceptive effects in the CNS (Figure 2; Ivashkiv and Donlin, 2014; Michalska et al., 2018; Tan et al., 2021; Wu et al., 2022).

### Regulatory mechanism of STING–IFN-I pathway

Regulation of the STING–IFN-I pathway mostly depends on STING activity. Posttranslational modifications including phosphorylation, ubiquitination, and palmitoylation play vital roles in regulating STING activity (Zhang H. et al., 2020). Activation of STING requires palmitoylation in the Golgi complex (Mukai et al., 2016; Haag et al., 2018). After recruiting TBK1 and activating IRF3, negative feedback is triggered. STING is subsequently phosphorylated by serine/threonine UNC-51-like kinase, and IRF3 activity is inhibited (Konno et al., 2013).

Among posttranslational modifications, ubiquitination is essential for STING activity. Some molecules have been shown to play essential roles in STING regulation. AMFR facilitates K27-linked polyubiquitination of STING through the ER membrane protein INSIG1 and promotes the recruitment and activation of TBK1 (Wang et al., 2014). EIF3S5, OTUD5, CYLD, and ubiquitin-specific protease (USP) 44 (USP44) are deubiquitinases that remove K48-linked polyubiquitination to maintain the stability of STING (Luo et al., 2016; Zhang et al., 2018; Zhang H. Y. et al., 2020; Guo et al., 2021). TRIM32 promotes K63-linked polyubiquitination of STING and increases the production of IFN-I (Cui et al., 2017). iRhom2 recruits the translocon-associated protein (TRAPβ) and the deubiquitination enzyme (EIF3S5) to promote STING trafficking from the ER to perinuclear microsomes (Luo et al., 2016). USP13 deconjugates polyubiquitin chains on STING to prevent recruitment of TBK1 (Sun et al., 2017), while USP21 hydrolyzes the K27/63-linked polyubiquitin chain on STING to negatively regulate the production of IFN-I (Table 1; Chen Y. et al., 2017). The regulatory mechanism of STING is complex and warrants further investigation.

**TABLE 1:** Summary of molecules associated with ubiquitination of STING and regulation of STING activity.

Molecules	Mechanism	Function
AMFR	Facilitates K27-linked Polyubiquitination through INSIG1	Promotes recruitment and activation of TBK1
EIF3S5	Remove K48-linked Polyubiquitination	Maintain stabilization of STING
OTUD5		
CYLD		
USP44		
TRIM32	Promotes K63-linked Polyubiquitination	Increases production of IFN-I
iRhom2	Recruits translocon-associated Protein TRAPβ and EIF3S5	Promote transmitting of STING from ER to perinuclear microsomes and maintain stabilization of STING
USP13	Deconjugates polyubiquitin chains on STING	Prevent recruitment of TBK1
USP21	Hydrolyzes K27/63-linked Polyubiquitin chain on STING	Decreases production of IFN-I

AMFR, EIF3S5, OTUD5, CYLD, USP44, TRIM32, and iRhom2 are positive regulators. USP13 and USP21 are negative regulators.

## Effects of STING–IFN-I pathway on nociception

Limited studies indicated that the STING–IFN-I pathway has dual effects on nociception (Table 2).

**TABLE 2:** Studies on the STING–IFN-I pathway and pain.

References	Study design	Animal/ Population	Model	Reagent injected	Injection method	Testing time	Mechanism (location)	Results	Effect
Donnelly et al. (2021)	Animal experiment	C56BL/6 mice	Chemotherapy-induced peripheral neuropathy model Nerve injury model Bone cancer pain model	DMXAA (STING agonists)	Intrathecal injection of 35 nmol DXMAA on day 0, day 3, day 6, day 9, and day 12	Tests conducted 4h after each injection	Activated STING-IFN-I pathway Restrained activity of ion channels Inhibit excitability of nociceptors (DRG/PNS)	Activation of STING-IFN-Ipathway in sensory neurons was sufficient to induce antinociception (DRG)	Positive
Wang et al. (2021)	Animal experiment	C56BL/6 mice	Lewis lung carcinoma cells induced bone cancer pain	DMXAA (STING agonist)	20 mg/kg injected intraprtitoneally twice on day 3 and day 7 after inoculation	Tests conducted 10 and 14 days after LLC inoculation	Activated STING-IFN-I pathway Inhibited osteoclastogenesis Reduced tumor burden (DRG/PNS)	Activation of STING-IFN-I pathway attenuated bone cancer pain	Positive
Sun et al. (2022)	Animal experiment	C57BL/6 male mice aged between 8 and 12 weeks	Chronic constriction injury	H-151 and 7-BIA	Intrathecal injection of 10 nM H-151 on day 7 after CCI Intraperitoneal injection of 7-BIA (10 or 20 mg/kg) on day 7 after CCI	Tests conducted 1.5, 6, 24, and 48 h after injection	Lack of protein tyrosine phosphatase receptor type D Activation of STING-IFN-I pathway (DRG/PNS)	Knockdown of protein tyrosine phosphatase receptor type D attenuated neuropathic pain *via* STING-IFN I pathway	Positive
Barragán-Iglesias et al. (2020)	Animal experiment	Male eIF4E^S209A^ and MNK1^–/–^ mice, C57BL/6J wild-type (WT) mice aged 8 and 12 weeks	Viral infection	IFN-α and IFN-β	Intraplantar administration of IFN-α (300 U/25 μl) or IFN-β (300 U/25 μl)	Tests conducted 1 h, 3 h, 24 h, 3 days, 6 days, 10 days after injection	Activation of MNK-eIF4E pathway Nociceptor hyperexcitability Mechanical pain sensitization (DRG/PNS)	Peripheral administration of IFN-I induced pain behavior in rats model in a short-term	Negative
Wu et al. (2022)	Animal experiment	Adult male Sprague–Dawley rats (200–220 g)	Spared nerve injury	RU.521 and C-176 (STING antagonist)	Consecutively intrathecal injection of 10 μM RU.521 and 5 μM C-176 on days 7–11 after SNI	Tests conducted 6 h after each injection	Activation of spinal cGAS/STING pathway Microglial M1-polarization (spinal cord/CNS)	Inhibition of cGAS-STING pathway suppressed microglial M1-polarizarion in the spinal cord and attenuated neuropathic pain	Negative
Liu et al. (2022)	Animal experiment	Male Sprague Dawley (SD) rats (180–230 g)	Spinal nerve ligation	2′3′-cGAMP (STING agonist) Ketamine; Dexmedetomidine	Intrathecal injections of 10 μg 2′3′-cGAMP on days 2, 4, and 6 after operation Intraperitoneal injection of 20 mg/kg ketamine and 20 μg/kg dexmedetomidine on postoperative days 2, 4, and 6	Tests conducted on days 3, 5, 7 after operation	Activation of STING/TBK pathway Inhibition of ER-phagy Enhancement of ER stress (Spinal cord/CNS)	Dexmedetomidine and ketamine attenuated neuropathic pain *via* STING pathway to induce ER-phagy	Negative
Lin et al. (2020)	Clinical trial (Prospective study)	372 HCV patients		Combinatory antiviral therapy (IFN-α-2beta + ribavirin)	1.5 μg of peg IFN-α-2beta per kilogram of body weight subcutaneously once weekly, and 600–800 mg of ribavirin daily for 24 weeks	Neurotoxicity Rating Scale (NRS) for somatic symptoms at baseline and at the 2nd, 4th, 8th, 12th, 16th, 20th, and 24th week	/	IFN-α therapy induces significant somatic pain symptoms as early as the 2nd week of treatment in HCV patients	Negative
Papa et al. (2021)	Clinical trial (Case series)	11 pediatric patients		Patients with COVID-19-related skin lesions	Paracetamol	age- and weight-adjusted paracetamol 15 mg/kg per dose, to a maximum of 750 mg per dose, every 6 – 8 h, with a maximum of 3,000 mgs daily for 10 days	/	In young patients, the IFN-1 response induces microangiopathic changes and produces a chilblain LE-like eruption with vasculitic neuropathic pain features	Negative

STING–IFN-I pathway has complex effects in different neuropathic pain models, effective time, and location of nerve system (see “Supplementary Appendix” for the search flow, method, and results).

### Positive effect

The STING–IFN-I pathway is associated with acute antinociceptive effects. In a rat model, deficiency of the STING–IFN-I pathway increased the excitability of nociceptors (Donnelly et al., 2021). In the chronic constriction injury model of rats, knockdown of the D-type protein tyrosine phosphatase receptor increased the expression of STING and IFN-α, which attenuated pain (Sun et al., 2022). STING agonists can relieve neuropathic pain in peripheral neuropathy induced by paclitaxel chemotherapy (Donnelly et al., 2021) and pain induced by nerve injury (Donnelly et al., 2021). Similarly, they may inhibit bone cancer pain and maintain motor function by reducing tumor burden and inhibiting cancer-induced osteoclast generation (Donnelly et al., 2021; Wang et al., 2021). Moreover, STING agonists can attenuate fracture-induced pain in tumor-free mice (Wang et al., 2021). Notably, after injecting STING agonists in rats, IFN-I levels in serum, DRG tissues, and bone marrow lysates were significantly upregulated 1000-fold in 4 h and maintained for up to 24 h (Donnelly et al., 2021; Wang et al., 2021; Sun et al., 2022). Therefore, the STING–IFN-I pathway may promote short-term antinociception.

### Negative effect

Several studies have also reported contradictory results, wherein the STING–IFN-I pathway exerted negative effects. A case series reported that IFN-I induced by STING causes neuropathic pain in young patients (Papa et al., 2021). Similarly, in patients with hepatitis C virus infection, the use of IFN-α leads to somatic pain (Lin et al., 2020).

Intraplantar administration of IFN-α (300 U/25 μL) or IFN-β (300 U/25 μL) can activate the MNK-eIF4E pathway *via* the STING–IFN-I pathway (Barragán-Iglesias et al., 2020). Subsequently, this pathway induces nociceptor hyperexcitability and mechanical pain sensitization at the DRG level for a short period of time (Barragán-Iglesias et al., 2020). Pain induction was not significant 3 days after peripheral injection (Barragán-Iglesias et al., 2020). Thus, the effects of IFN-I may be acute or transient.

In the spared nerve injury (SNI) model, inhibiting the cGAS–STING pathway can restrain microglial M1-polarization and attenuate neuropathic pain (Wu et al., 2022). M1-polarization microglia express CD16 and induce TNF-α and IL-1β synthesis, which may cause central sensitization (Mesquida-Veny et al., 2021). In a rat SNL model, ketamine and dexmedetomidine induced ER-phagy and alleviated ER stress to provide antianxiety and antinociceptive effects by inhibiting the STING–TBK pathway in the spinal cord (Liu et al., 2022).

### Underlying reasons for the dual effects

**FIGURE 3:**
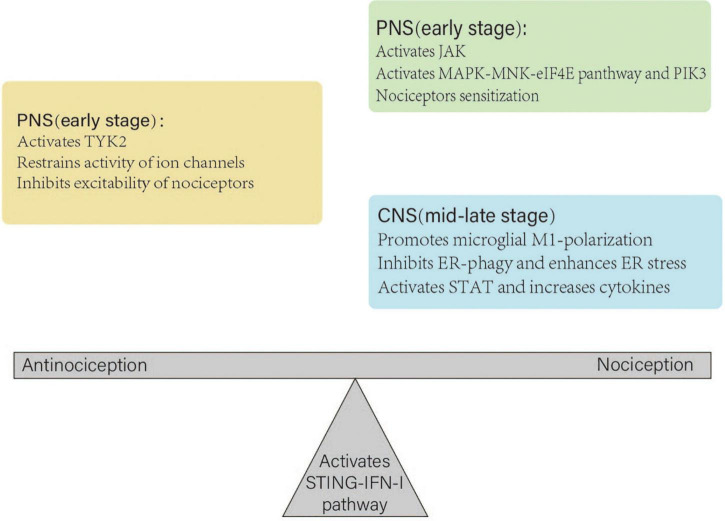
Different roles of the STING–IFN-I pathway in the peripheral nerve system (PNS) and central nerve system (CNS). In the PNS, the pathway inhibits the excitability of nociceptors at an early stage. Subsequently, it may cause nociceptor sensitization. At the mid-late stage, it plays a dominant role in the CNS, which leads to central sensitization. Under normal physiological conditions, antinociception and nociception of the STING–IFN-I pathway maintain homeostasis. A nerve injury disrupts the balance and leads to antinociception or nociception.

There are several possible explanations for these contradictions. First, the sex of the animals may have caused this discrepancy. This pathway more likely has a negative effect on male rats (Wu et al., 2022). Second, animal experiments were used to create different neuropathic pain models to explore its effects. However, different animal models may exhibit various neuropathies. Third, different injection methods may also have caused bias. Peripheral administration of IFN-I induced pain behavior in rat models (Barragán-Iglesias et al., 2020). However, intrathecal injection of IFN-α inhibited mechanical hypersensitivity caused by intraplantar (Donnelly et al., 2021). Fourth, the different effective times influenced the results. Short-term activation of this pathway led to transient and acute antinociception, which was maintained for up to 24 h (Wang et al., 2021). However, consecutive and repeated administration of STING agonists caused central sensitization and nociception (Wu et al., 2022). Fifth, the STING–IFN-I pathway does not participate in the physiological regulation of pain sensitivity and is only involved in the regulation of pain after nerve injury (Sun et al., 2022; Wu et al., 2022). Therefore, observing a positive effect in normal rat models injected with STING agonists or IFN-I is challenging. Finally, the STING–IFN-I pathway may play distinct roles in different parts of the PNS and CNS. A study has demonstrated that after STING agonist DMXAA treatment in mouse models, bone cancer-induced cold and mechanical allodynia were reduced at an early stage but not at the mid-late stage (Donnelly et al., 2021). Therefore, at an early stage, it induces antinociception and reduces pain by restraining the activity of ion channels and the excitability of nociceptors in the PNS. Subsequently, this pathway may induce nociceptor sensitization *via* the MAPK–MNK–eIF4E pathway and PIK3. At the mid-late stage, it can cause central sensitization in several ways (Figure 3).

## STING agonists compared with opiates

Since the isolation of morphine in 1805, opioids have been widely used for pain management (Pasternak, 2014). Opioids, including morphine, interact with μ, κ, and δ receptors to produce analgesic effects, respiratory depression, and euphoria addiction. After binding with opioid receptors, opioids cause antinociception through the same mechanism as enkephalin, which involves hyperpolarization of interneurons and reduction of transmitters associated with pain (Haigler, 1987; Lipp, 1991). In addition, morphine can react with opioid receptors in supraspinal structures to activate the supraspinal system (Lipp, 1991). By contrast, STING agonists produce acute and short-term antinociception *via* the STING–IFN-I pathway in the PNS. Furthermore, opioids are highly addictive, which is caused by a reduction in the inhibitory function of GABAergic synapses in the neurons of the central amygdala and brain reward/motivational mesocorticolimbic circuitry (Navratilova and Porreca, 2014; Zhang et al., 2014). In contrast, the repeated use of STING agonists does not cause addiction and attenuates SNI-induced astrogliosis (Donnelly et al., 2021). In non-human primates, intrathecal administration of STING agonists produces longer lasting analgesic effects at lower doses than morphine (3 vs. 100 nmol) (Sjöström et al., 1987; Donnelly et al., 2021; Wang et al., 2021). Naloxone, a nonselective and short-acting opioid receptor antagonist, can reverse the analgesic effect of morphine (Drug and the Therapeutics Bulletin, 1981; van Dorp et al., 2007). By contrast, STING agonist-mediated analgesia is not affected by naloxone (Donnelly et al., 2021).

Previous studies have suggested that STING agonists have potential advantages including strong efficacy at low doses, a longer lasting effect, and non-addictive. However, the exact effects of STING–IFN-I on nociception remain unclear and require further investigation.

## Discussion

Apart from inducing antimicrobial response, mediating autoimmune disease, and regulating tumor growth, the STING–IFN-I pathway can induce acute antinociception for a short period of time. Therefore, the STING–IFN-I pathway may be a potential therapeutic target for pain management.

However, the effects of STING on nociception have several issues that need to be discussed. First, Donnelly et al. (2021) demonstrated that STING agonists reduced bone cancer pain. However, Zhang et al. (2022) suggested that mitochondrial DNA triggers the STING pathway, leading to peripheral neuroinflammation and sensitization (Zhang et al., 2022). In the early stage, the STING–IFN-I pathway was dominant, which reduced bone cancer pain. In the mid-late stage, the MAPK–MNK–eIF4E pathway was activated, and the STING–NF-κB pathway increased bone cancer pain *via* IL-1β, IL-6, and TNF-α (Barragán-Iglesias et al., 2020; Zhang et al., 2022). In addition, STING agonists have been shown to reduce bone cancer pain through immune and neuronal modulation, reducing tumor burden and inhibiting osteoclastogenesis (Amouzegar et al., 2021; Wang et al., 2021). Therefore, it is difficult to determine the true effects of STING agonists in bone cancer pain models. Second, the STING–IFN-I pathway may influence central sensitization through ER-phagy and microglial M1-polarization. Further studies are needed to confirm this hypothesis and to determine how the STING–IFN-I pathway regulates ER-phagy and microglial M1-polarization. Third, previous studies have only discussed one downstream pathway in a neuropathic pain model. However, STING has various downstream signaling components, including IFN-I, NF-κB, and NLPRS. Studies that include all downstream signals of STING are still lacking. Lastly, STING–IFN-I exists not only in peripheral and central neurons but also in immune cells. Whether STING agonists interact with these cell types to cause nociception requires further studies (Wang et al., 2021).

The current clinical use of STING agonists focuses on cancer immunotherapy. Several combination therapies are currently available in clinical trials (Zheng et al., 2020). Few studies have indicated the effectiveness of STING in nociception; however, the use of STING for nociception remains controversial and warrants further extensive and comprehensive studies.

## Conclusion

At an early stage, the STING–IFN-I pathway can induce short-term antinociceptive effects by activating TYK2, restraining the activity of calcium and sodium channels, and inhibiting the excitability of nociceptors in the PNS. Subsequently, it activates the JAK–MAPK–MNK–eIF4E pathway and PIK3, which cause nociceptor sensitization. At the mid-late stage, it promotes microglial M1-polarization, inhibits ER-phagy, activates STAT, and increases the expression of pro-inflammatory and anti-inflammatory cytokines in the CNS, which leads to central sensitization. Thus, the STING–IFN-I pathway at various stages has a dual effect on nociception.

## Author contributions

JY wrote the manuscript and made illustrations. HD, BS, and YZ (Fifth author) provided advice for the manuscript. YZ (Fourth author) provided the supervision and comments on the manuscript. All the authors read and approved the final manuscript.

## Supplementary material

Supplementary Data Sheet 1 Search process for cited articles. Forty-seven articles were found initially. After excluding ineligible articles, only eight articles were included.
