# Supplementary material for: Mechanism and effects of STING–IFN-I pathway on nociception: A narrative review
Source: Front Mol Neurosci. 2023 Jan 4;15:1081288. doi: 10.3389/fnmol.2022.1081288 (PMC9846240; doi:10.3389/fnmol.2022.1081288)

**Appendix**

**Search Flow of PubMed Database**

((((((((((("stimulator of interferon genes ") OR ("STING1 protein, human")) OR ("mediator of IRF3 activation protein, human")) OR ("MPYS protein, human")) OR ("STING protein, human")) OR ("transmembrane protein 173, human")) OR ("MITA protein, human")) OR ("stimulator of interferon response cGAMP interactor 1 protein, human")) OR ("Tmem173 protein, human")) OR ("STING-beta protein, human")) OR ("ERIS")) AND ((((((("Nociception") OR ("Nociceptions")) OR ("Nociperception")) OR ("Nociperceptions")) OR ("Pain")) OR ("Ache")) OR ("Aches"))

**Search Flow of Web of Science Database**

#1 (((((((((TS=("STING1 protein, human")) OR TS=("mediator of IRF3 activation protein, human")) OR TS=("MPYS protein, human")) OR TS=("STING protein, human")) OR TS=("transmembrane protein 173, human")) OR TS=("MITA protein, human")) OR TS=("stimulator of interferon response cGAMP interactor 1 protein, human")) OR TS=("Tmem173 protein, human")) OR TS=("STING-beta protein, human")) OR TS=("ERIS")

#2 ((((((TS=("Nociception")) OR TS=("Nociceptions")) OR TS=("Nociperception")) OR TS=("Nociperceptions")) OR TS=("Pain")) OR TS=("Ache")) OR TS=("Aches")

#3 (#1) AND #2

**Search Flow of Embase Database**


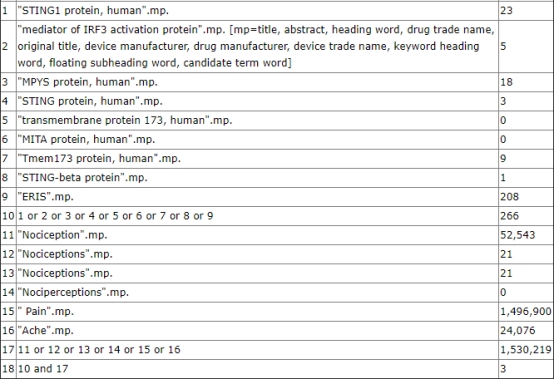


**Search Results**


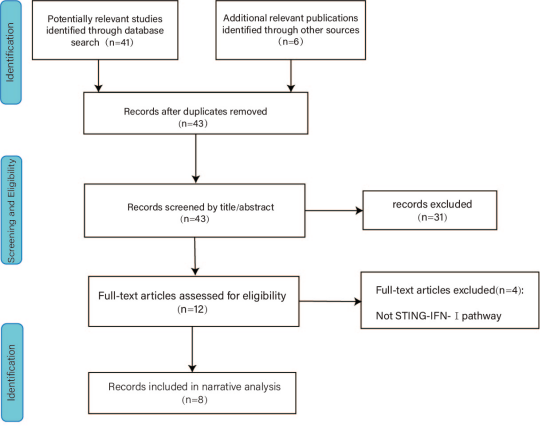

Supplement: Supplementary Data Sheet 1 — Search process for cited articles. Forty-seven articles were found initially. After excluding ineligible articles, only eight articles were included. [file Data_Sheet_1.docx]
